# BUViTNet: Breast Ultrasound Detection via Vision Transformers

**DOI:** 10.3390/diagnostics12112654

**Published:** 2022-11-01

**Authors:** Gelan Ayana, Se-woon Choe

**Affiliations:** 1 Department of Medical IT Convergence Engineering, Kumoh National Institute of Technology, Gumi 39253, Korea; 2 Department of IT Convergence Engineering, Kumoh National Institute of Technology, Gumi 39253, Korea

**Keywords:** breast cancer, ultrasound, vision transformer, convolutional neural network, transfer learning

## Abstract

Convolutional neural networks (CNNs) have enhanced ultrasound image-based early breast cancer detection. Vision transformers (ViTs) have recently surpassed CNNs as the most effective method for natural image analysis. ViTs have proven their capability of incorporating more global information than CNNs at lower layers, and their skip connections are more powerful than those of CNNs, which endows ViTs with superior performance. However, the effectiveness of ViTs in breast ultrasound imaging has not yet been investigated. Here, we present BUViTNet breast ultrasound detection via ViTs, where ViT-based multistage transfer learning is performed using ImageNet and cancer cell image datasets prior to transfer learning for classifying breast ultrasound images. We utilized two publicly available ultrasound breast image datasets, Mendeley and breast ultrasound images (BUSI), to train and evaluate our algorithm. The proposed method achieved the highest area under the receiver operating characteristics curve (AUC) of 1 ± 0, Matthew’s correlation coefficient (MCC) of 1 ± 0, and kappa score of 1 ± 0 on the Mendeley dataset. Furthermore, BUViTNet achieved the highest AUC of 0.968 ± 0.02, MCC of 0.961 ± 0.01, and kappa score of 0.959 ± 0.02 on the BUSI dataset. BUViTNet outperformed ViT trained from scratch, ViT-based conventional transfer learning, and CNN-based transfer learning in classifying breast ultrasound images (*p* < 0.01 in all cases). Our findings indicate that improved transformers are effective in analyzing breast images and can provide an improved diagnosis if used in clinical settings. Future work will consider the use of a wide range of datasets and parameters for optimized performance.

## 1. Introduction

Breast cancer is the most prevalent malignancy, accounting for 12.5% of new cases globally by 2020 [1,2]. The survival rate of breast cancer for at least five years after diagnosis varies among populations depending on the economic standing of nations; it is greater than 90% in high-income nations, 40–65% in middle-income nations, and less than 40% in low-income nations [2,3]. This difference is primarily attributable to the ability of high-income nations to detect breast cancer early [1]. Early detection expands treatment options and lowers the risk of breast-cancer-related deaths considerably. The primary imaging modalities for the early diagnosis of breast cancer include mammography and ultrasound [4].

Previously, ultrasonography was considered helpful only for cyst diagnosis. However, it improves local preoperative staging, guided interventional diagnosis, and differential diagnosis of benign and malignant tumors [5]. Mammography has a low sensitivity for thick breasts [6]. Women with dense parenchyma are far more likely to develop breast cancer [7]. Dense breast tissue can be examined using ultrasound [8]. Recent research has demonstrated that high-resolution ultrasonography increases the diagnosis of tiny tumors by three to four tumors per 1000 women without clinical or mammographic abnormalities [9]. Carcinomas observed on mammography and sonography have a similar stage distribution [10]. To overcome the limitations of mammography, ultrasound is frequently employed for curative diagnosis [11]. Ultrasound is noninvasive, widely available, easy to use, less expensive, and provides real-time imaging [12]. Moreover, ultrasound is safer and does not generate radiation [13]. More importantly, ultrasound helps detect tumors in women with dense breasts and detects and classifies breast lesions that cannot be interpreted adequately through mammography alone [14]. Doctors can use ultrasound to identify many clinically relevant regions such as benign cysts or normal tissues. Most women over the age of 30 years usually undergo ultrasonography along with mammography. For women under the age of 30, ultrasonography is sufficient to decide whether a biopsy is necessary in a particular area of the suspected breast.

However, early breast ultrasound diagnosis has limitations [15]. First, follow-up ultrasound, aspiration, or biopsy may be performed after a breast ultrasound image is interpreted [16]. Biopsy may be recommended to determine whether a suspicious abnormality is cancer. Most of the suspected problem locations detected by ultrasonography that require biopsy are noncancerous (false positives) [17]. Second, although ultrasound is a breast imaging method, annual mammography is still recommended [1]. Many tumors cannot be detected via ultrasonography. Numerous calcifications detected by mammography are invisible with ultrasonography [18]. In mammography, some early breast tumors simply manifest as calcifications [19,20]. Even for women with dense breasts, many institutions do not offer ultrasound screening, and some insurance plans might not pay for treatment. As a real-time test, ultrasound depends on the anomaly detected during the scan. Therefore, sophisticated tools and experienced professionals are required.

Deep learning (DL) has been used to overcome the limitations of ultrasound in the early detection of breast cancer [21]. Many studies have been conducted in the areas of synthetic imaging, object detection, segmentation, and imaging categorization of breast lesions using DL [22]. Many of these approaches have received the necessary legal certifications and are used in clinical settings [23]. Additionally, DL approaches have demonstrated ability to perform at levels comparable to those of human experts in a variety of breast cancer detection tasks and have the potential to help physicians with little training improve the diagnosis of breast cancer in clinical settings [24].

All prior investigations regarding DL for ultrasound-based early breast cancer diagnosis have used convolutional neural networks (CNNs) [25]. Vision transformers (ViTs), developed by Dosovitskiy et al. [26] in 2020, outperform state-of-the-art (SOTA) CNN algorithms in natural images categorization. ViTs demonstrate a superior performance by incorporating more global information than a CNN does in lower layers and having a stronger skip connection than that of ResNet. CNNs require several layers to calculate features computed by a smaller collection of lower layers of ViTs. All these attributes help ViTs outperform CNNs in classifying real-world images. However, not much research has been conducted on the application of ViTs in medical image analysis. This is mainly because of the heavy reliance of ViTs on extensive training data. ViTs do not outperform CNNs with small-scale datasets. The heavy reliance of ViTs on big data has limited its effective use in certain sectors. The same is true for medical images, as it can be difficult to locate substantial training datasets. To overcome this challenge, transfer learning has been extensively used in CNN-based medical image analysis [25,27,28]. The use of ViT models that have been pretrained on large natural image datasets and transfer learning via ViT models may help increase the performance of DL in medical image analysis. Hence, we present BUViTNet breast ultrasound detection via ViTs, a new transfer-learning strategy based on ViTs for the classification of ultrasound breast cancer images.

The proposed ViT-based transfer learning has multiple advantages over CNN-based transfer learning. The disadvantages of CNNs include their high processing cost, narrow focus on a portion of the image rather than the entire image, inability to encode relative spatial information, and inability to handle rotation and scale invariance without augmentation. CNNs are computationally expensive owing to the use of pixel arrays [29]. Because deeper layers are required to extract features from the entire image, CNNs require more training time and hardware. In addition, because of the information lost during processes such as pooling (average- or max-pooling), CNNs become slower and less accurate. A convolutional layer is the primary building block of a CNN. Its task is to identify significant details in the image pixels. Higher layers combine simple characteristics into more complex features, whereas deeper layers (closer to the input) learn to recognize simple features such as edges and color gradients. Very high-level features are finally combined in thick layers at the top of the network to create classification predictions. All higher-level neurons in a CNN receive low-level information. Then, these neurons perform additional convolutions to determine whether specific features are present. This is accomplished by repeating the knowledge across all the individual neurons after traversing the receptive field. The location and orientation of the object are not considered by a CNN when forming the predictions. They entirely lose all internal information about the position and orientation of the item and route all of the data to the same neurons that may not be able to process this type of input [30]. A CNN predicts output by observing an image and determining whether specific elements are present. If they are, the image is appropriately categorized. An artificial neuron produces only one scalar. Additionally, CNNs employ convolutional layers that produce a 2D matrix, with each number representing the result of the kernel’s convolution with a portion of the input volume. These layers duplicate the weights of each kernel across the entire input volume for each kernel. Therefore, we can consider the 2D matrix as the output of the replicated feature detector. The output of a convolutional layer is then produced by stacking all the 2D matrices of the kernel on top of one another. The next step is to attempt to attain perspective invariance in the neuronal activity. This is accomplished using max pooling, which selects the greatest value in each region of the 2D matrix after looking at each region in turn. Consequently, invariance is achieved. When the output is invariant, the input can be slightly altered without affecting the results. In other words, max pooling ensures that the network activities (neuron outputs) remain constant even if the object we wish to detect is slightly shifted in the input image, allowing the network to continue to recognize the object. However, because max pooling discards important data and does not encode the relative spatial relationships between features, the approach described above is ineffective [31]. Consequently, CNNs are not genuinely immune to significant input data alterations. The use of ViTs enables us to overcome these limitations of CNNs to detect breast cancer using ultrasound images. In this study, we also introduced a novel transfer-learning method to compensate for the ViT’s data hunger. The use of transfer learning enables ViT to be pretrained on a large number of natural and microscopic images and to be used to classify ultrasound images without overfitting.

Generally, the proposed method provides the following contributions.

- Developed the first multistage transfer-learning method using vision transformers for breast cancer detection.
- Utilized microscopic image datasets that have related image features to those of ultrasound images for intermediate-stage transfer learning to improve the performance of breast cancer early detection.
- Carefully studied the characteristics of different vision transformers based on pretrained models for translation to ultrasound image-based breast cancer detection.
- Investigated the effectiveness of the proposed BUViTNet method when applied to datasets from different sources as well as on mixed datasets from different origins.
- Compared the performance of the BUViTNet method against vision transformers trained from scratch, conventional vision transformer-based transfer learning, and convolutional neural networks for breast cancer detection.

## 2. Materials and Methods

### 2.1. Dataset

We evaluated our method using two publicly available datasets: breast ultrasound image (BUSI) (Available online: https://scholar.cu.edu.eg/?q=afahmy/pages/dataset (access on 1 July 2022)) and Mendeley breast ultrasound (Available online: https://data.mendeley.com/datasets/wmy84gzngw/1 (access on 1 July 2022)) datasets. Moreover, we created a mixed BUSI and Mendeley dataset to evaluate the performance of the proposed method on datasets from different sources.

**Figure 1:**
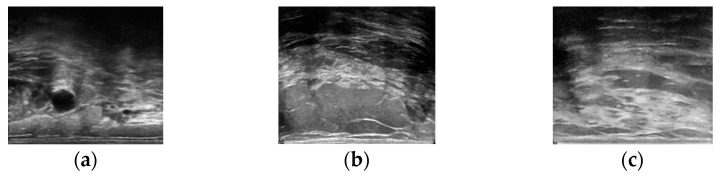
Representative images from BUSI dataset: (**a**) Benign; (**b**) Malignant; (**c**) Normal.

The BUSI dataset is categorized into three classes: benign (210 images), malignant (437 images), and normal (210 images). BUSI dataset images were taken from women between the ages of 25 and 75 years; hence, the dataset is preferred for studies involving early breast cancer detection in women below 40 years of age [32]. The dataset was collected in 2018 from 600 female patients. The dataset consists of 780 images, each with an average size of 500 × 500 pixels. The dataset images are PNG files. Representative images from the dataset are shown in Figure 1.

**Figure 2:**
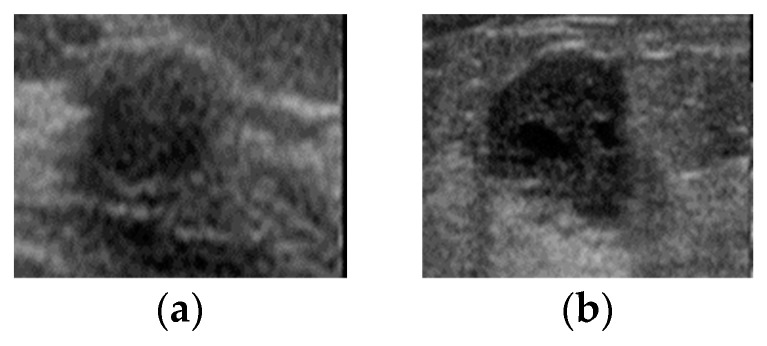
Representative images from Mendeley breast ultrasound dataset: (**a**) benign; (**b**) malignant.

The Mendeley breast ultrasound dataset is divided into two categories: benign (100 images) and malignant (150 images) [33]. The dataset was collected in Brazil from women aged 30–70 years. The images in the dataset are in PNG format and of different sizes. This dataset is practical for our needs and was extensively used in numerous investigations. Representative images from this dataset are shown in Figure 2.

The mixed dataset is categorized into two classes: benign (310) and malignant (587). We mixed the datasets to evaluate the performance of the proposed method on datasets from different locations. We added benign images from the BUSI and Mendeley datasets to make a mixed benign image class, and the same was done for malignant images.

### 2.2. Preprocessing

**Figure 3:**
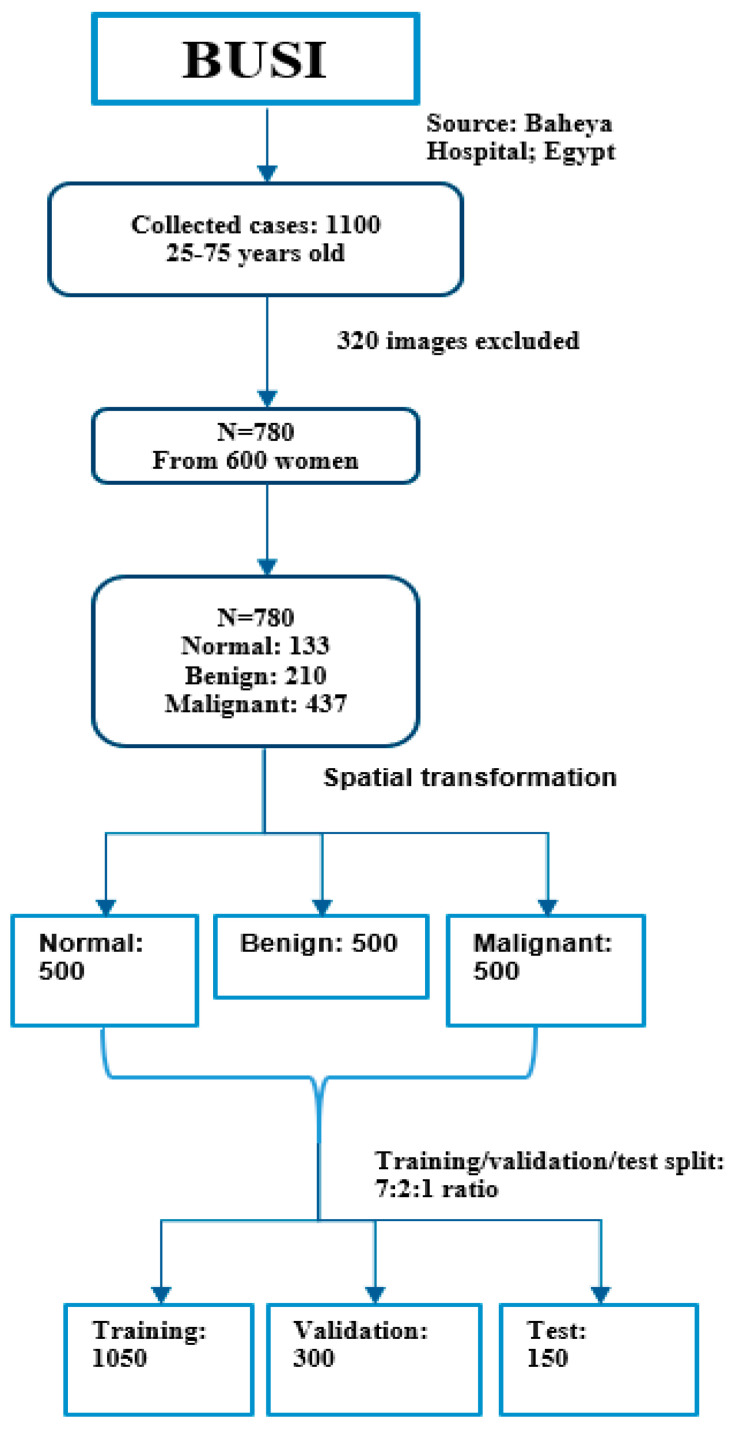
Dataset distribution after preprocessing the BUSI dataset.

The preprocessing of the data involved resizing the dataset images and balancing the number of images in each class. The images in both datasets utilized in this study had different pixel sizes from their corresponding sources. Therefore, we resized them to use them as input in our proposed method. Each image was resized to 224×224 pixels. Additionally, the datasets were class-imbalanced owing to the different number of images for each class. Class imbalance impinges upon the learning process by biasing the model toward the class having a larger dataset size. To prevent this, we implemented spatial transformation-based augmentations (horizontal flip, color jitter, sharpening, salt-and-pepper, gamma correction, random rotation, and height shift) to equalize the size of the images in each class. Subsequently, we created a BUSI dataset with three classes, each having 500 images; a Mendeley dataset with two classes, each having 500 images; and a mixed dataset with two classes, each having 1000 images. Following this, we categorized our dataset into training, validation, and test sets with a 7:2:1 ratio. Figure 3 depicts the dataset distribution after preprocessing the BUSI dataset. The training data were further augmented to enrich the training images. We performed random rotation, flip (both horizontal and vertical), shift (both width and height), shear, and other augmentations.

### 2.3. Proposed Method

**Figure 4:**
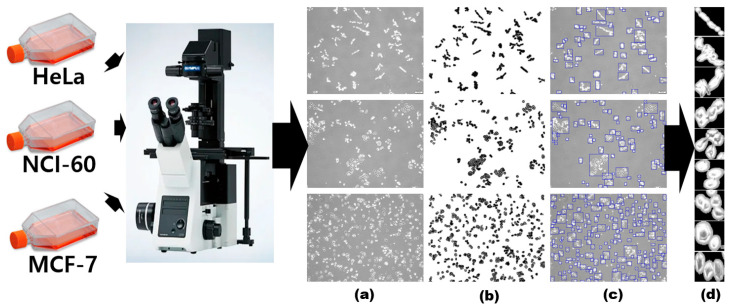
Acquisition and preprocessing of cancer cell line images: (**a**) acquired image (**b**) binarized image (**c**) patch selection (**d**) final cancer cell images used for training. MCF-7, breast cancer cell; NCI-60, lung cancer cell; HeLa, cervical cancer cell.

The proposed method employs multistage transfer learning. A ViT model pretrained using the ImageNet dataset was first used for transfer learning with cancer cell line images to distinguish breast (MCF-7), lung (NCI-60), and cervical (HeLa) cancers. Figure 4 shows the acquisition and preparation of the cancer cell images for the intermediate stage transfer learning. The cancer cell images were captured under microscope, separated from the background, and then segmented to form patches of each cancer cell line. Details about acquisition and preprocessing of cancer cell lines can be found in [28]. Using the ImageNet pretrained model, transfer learning was employed to train a model for classifying the cancer cell line images as breast, lung, and cervical cancers.

**Figure 5:**
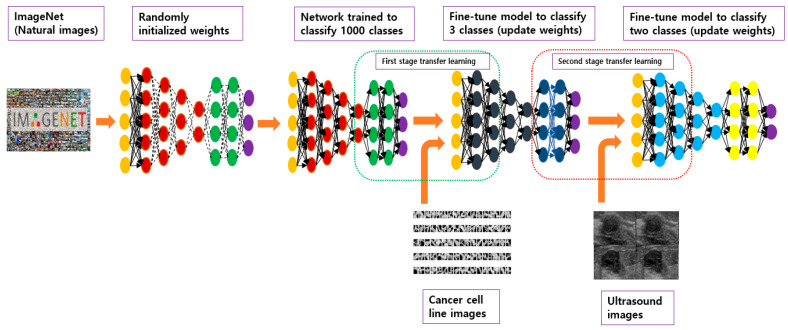
The proposed transfer-learning approach using the mixed ultrasound dataset.

Then, the model trained for cancer cell classification was used as a pretrained model for transfer learning on breast ultrasound images. Figure 5 depicts the multistage approach used in this study. Multistage transfer learning was applied for two main reasons. The first was to develop a model that was trained on large image datasets with features similar to those of ultrasound images, which can be acquired in large numbers and are ideal as intermediate-stage transfer learning domains. A previous study we conducted also proved that CNN models pretrained on cancer cell line images improved the performance of classifying breast ultrasound images. The second reason was that the use of cancer cell lines would optimize large ViT models, which are data-hungry, for small datasets. We utilized 28,560 cancer cell images [28] to train the ViT models in the intermediate transfer-learning stage before using them for classifying ultrasound images. 

Multistage transfer learning was applied using three ViT models: a base model with 16×16 input patch size (vitb_16), a base model with 32×32 input patch size (vitb_32), and a large model with 32×32 input patch size (vitl_32). Table 1 lists their details. 

**Figure 6:**
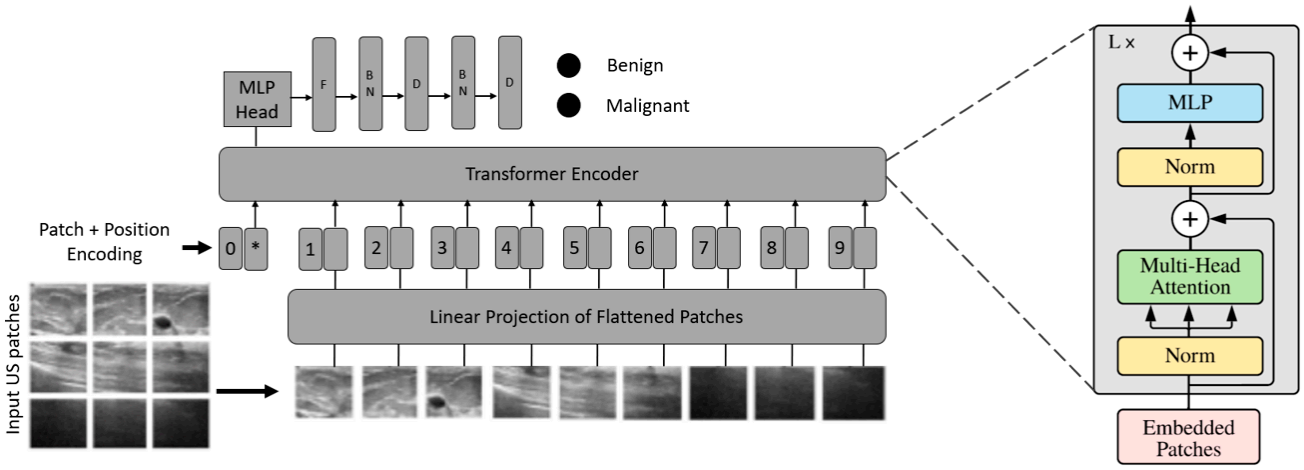
The proposed vision transformer-based transfer-learning model. US, ultrasound; MLP, multilayer perceptron; F, flatten; BN, batch normalization; D, dense layer.

The ViT-based transfer learning at both intermediate and task levels was implemented by replacing the last layer of each ViT model using a flattening layer followed by batch normalization, one dense layer followed by batch normalization, and a final dense layer as a classifier, as shown in Figure 6.

A transformer model is a neural network that learns context and meaning by tracking relationships in sequential data, such as the words in a sentence. Transformer models apply an evolving set of mathematical techniques, called attention or self-attention, to detect subtle ways by which even distant data elements in a series influence and depend on each other. ViTs use data sequences in the form of image patches. The ViTs used in the study operate as follows.

- Conversion of images into patches: ViTs use image patches as tokens of words, as in the original paper Vaswani et al. [34]. For images, pixels can be considered; however, it increases the computational cost. Moreover, finding hardware to process high-resolution images such as medical images is challenging. Therefore, we propose the conversion of input images into patches, as in [26]. In this study, an image with H×W was converted into N patches of size P×P.
- Flattening and patch embedding: The patches were then flattened and sent through a single feed-forward layer to obtain a linear patch projection. This feed-forward layer contained the embedding matrix E, as mentioned in Dosovitskiy et al. [26]. Matrix E is randomly generated.
- Learnable embedding and positional embedding: Learnable embeddings are concatenated with patch projection, which is used later for classification. Because patches are not naturally formed in sequences, as in time sequence models, the transformers utilized positional embeddings to establish a certain order in the patches. The positional encoding matrix is randomly generated, as in patch embedding.
- Multilayer perceptron (MLP) head: The outputs of the transformer encoder unit are passed to the MLP head for classification. Despite the multiple outputs from the transformer encoder, the MLP considered only one output related to the class embedding, whereas the other outputs are ignored. MLP outputs the probability distribution of the labels to which the corresponding images belong.

### 2.4. Implementation Details

We trained our models for 50 epochs using the Adam optimizer with a learning rate of 0.0001. A batch size of 16 and exponential decay were used for training. Our datasets were divided with a 7:2:1 ratio for training, validation, and testing. GELU was utilized as an activation function with an L2 regularizer for the ViT models. In CNNs, ReLu was utilized with an L2 regularizer. For experiments involving comparison with the proposed method, the same parameter settings were utilized to avoid bias in the results.

### 2.5. Experimental Settings

Four experiments were conducted to evaluate the proposed model. The first experiment was conducted to compare the performance of the proposed model in three ViT models (vitb_16, vitb_32, and vitl_32). This was done to select the best-performing model having the least computational cost. In the second experiment, a ViT model trained from scratch was implemented and compared with the proposed transfer-learning model. This experiment evaluated whether the ViT trained from scratch was more suitable for classifying breast ultrasound images than a ViT pretrained on different datasets prior to being applied to the task of classifying breast ultrasound images. The third experiment was performed to choose the transfer-learning approach (conventional ImageNet-only pretraining or the proposed transfer-learning method) appropriate for the ViT-based transfer- learning method in classifying breast ultrasound images. Finally, in the fourth experiment, the performance of ViT-based transfer learning was evaluated and compared with that of CNN-based transfer learning. This experiment provides an effective comparison between ViT-based and CNN-based transfer-learning methods for breast ultrasound image classification.

### 2.6. Evaluation Metrics

Metrics including accuracy, area under the receiver operating curve (AUC), F1-score, recall, and precision were used to assess the performance of our model. We used Matthew’s correlation coefficient (MCC) and kappa scores also to assess the performance of our model, owing to the uneven nature of our dataset and the superiority of these metrics in analyzing the performance. A 95% confidence interval was used to determine each outcome. In Table 2, TP, TN, FP, and FN stand for true positive, true negative, false positive, and false negative, respectively.

## 3. Results

The proposed method was evaluated using two datasets from different sources and their mixture, as listed in Table 3. The proposed method achieved an AUC of 1 ± 0, MCC score of 1 ± 0, and kappa score of 1 ± 0 on the Mendeley dataset for the vitb_16, vitb_32, and vitl_32 ViT models. Furthermore, the proposed method achieved the highest AUC of 0.968 ± 0.02, MCC score of 0.961 ± 0.01, and kappa score of 0.959 ± 0.02 on the BUSI dataset for the Vitb _16 ViT model. For the mixed dataset, the vitb_16 model achieved the highest AUC of 0.937 ± 0.03, MCC score of 0.924 ± 0.02, and kappa score of 0.919 ± 0.03.

**Figure 7:**
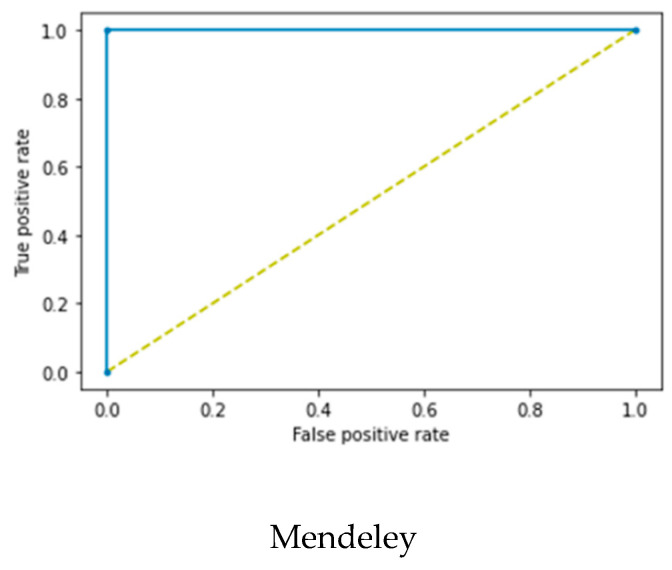

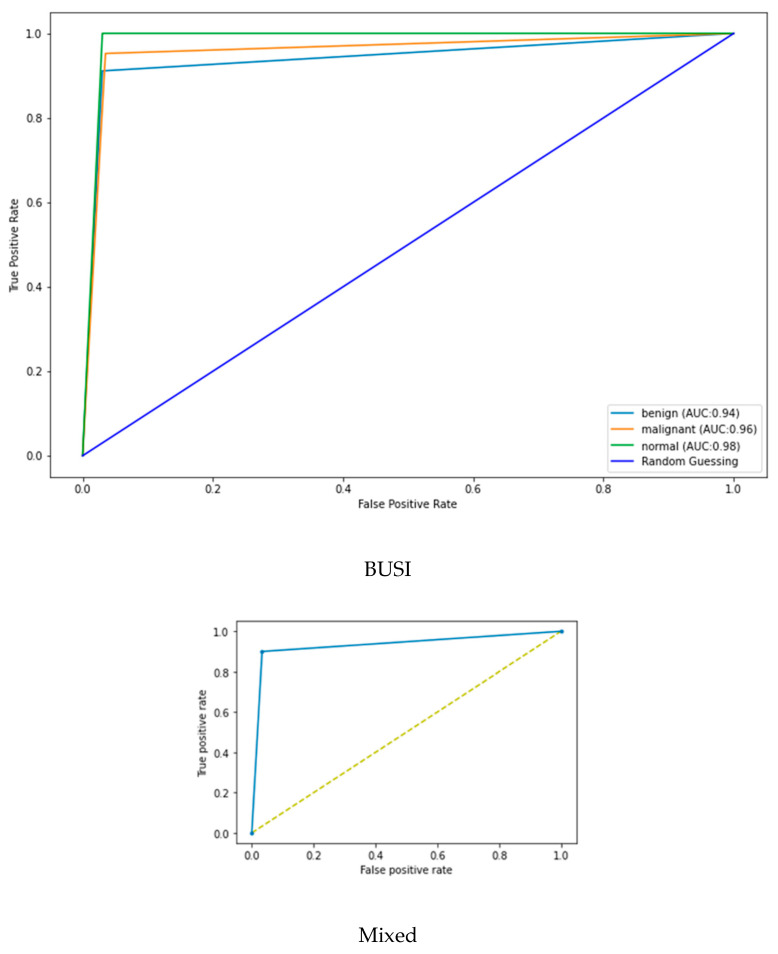
Receiver operating characteristics (ROC) curves of the proposed method on Mendeley, BUSI, and mixed datasets. BUSI, breast ultrasound image dataset.

Figure 7 shows the receiver operating characteristics (ROC) curves of the proposed BUViTNet method on the three datasets, using vitb_16 as a base model. 

We compared the proposed method with ViT models trained from scratch using ultrasound images. This was performed to determine whether the ViT-based transfer-learning model performed better than a ViT model trained directly from scratch with ultrasound images. The highest AUC recorded with the models trained from scratch were 0.73 ± 0.2, 0.71 ± 0.07, and 0.7 ± 0.1 using vitb_16 on Mendeley, BUSI, and mixed datasets, respectively (Table 4). The proposed method significantly outperformed the ViT models trained from scratch with a *p*-value of less than 0.01 in all cases on all datasets.

Furthermore, the proposed transfer-learning method was compared with conventional ImageNet-pretrained ViT models. This experiment validates the superiority of the proposed method over traditional transfer learning. The highest AUCs achieved for conventional transfer learning using ViT models were 1 ± 0, 0.9548 ± 0.0183, and 0.9116 ± 0.0156 on Mendeley, BUSI, and mixed datasets, respectively (Table 5). The performances of the proposed method and traditional transfer learning on the Mendeley dataset were comparable; however, the proposed method outperformed the traditional transfer-learning method on BUSI and mixed datasets, with a *p*-value of less than 0.01.

Finally, we compared the proposed ViT-based transfer-learning method with the transfer-learning method using CNNs. To do so, we used three SOTA CNN architectures: ResNet50, EfficientNetB2, and InceptionNetV3. All implementation parameters were kept the same as those of the ViT-based transfer-learning method for a fair comparison. ResNet50-based transfer learning provided the highest AUC scores of 0.972 ± 0.01, 0.879 ± 0.2, and 0.836 ± 0.08 on Mendeley, BUSI, and mixed datasets, respectively (Table 6). The proposed ViT-based transfer-learning method performed better than the CNN-based transfer-learning method for breast ultrasound images, with a *p*-value of less than 0.01.

## 4. Discussion

In this study, we proposed a novel transfer-learning approach for ViT-based breast ultrasound image classification. Our approach uses a ViT model pretrained on the ImageNet dataset for transfer learning to classify cancer cell images. This model, trained on ImageNet and cancer cell images, was then used to classify breast ultrasound images. This novel transfer-learning approach enables a model to learn from a large number of natural and medical images before being used for classifying breast ultrasound images. The model leverages the features learned in the previous transfer-learning stages to use it for the target task. As a result, we were able to achieve the best performance in terms of all metrics used with our proposed model. We compared the proposed approach with ViT models trained from scratch, ViT-based conventional transfer learning, and CNN-based transfer learning. The proposed approach outperformed all of these models.

Regarding the performance of BUViTNet using various base models, vitb_16-based BUViTNet performed better than vitb_32 and vitl_32, thereby providing less computational complexity. The main reason for this might be the size of the patches used in these base models. The vitb_16 base model utilizes an input patch size of 16 × 16, whereas vitb_32 and vitl_32 base models utilize an input patch size of 32 × 32. The smaller the patch size, the higher the efficiency and effectiveness of the transformer encoder’s attention. This leads to a better performance in extracting local and global features, which consequently improves tumor classification. The other reason, specially, in the case of vitl_32 is that the network is larger and overfits the data compared to that of vitb_16 and vitb_32 models. Moreover, the vitb_16 model is less computationally complex compared to vitb_32 and vitl_32 models, making it preferable in our case.

Despite the superior performance of the proposed method in all the experiments, it has some disadvantages. As can be observed from the results in Table 4, ViT trained from scratch performed poorly. This is because ViTs are data intensive. ViTs require a large number of images to perform well when trained from scratch, owing to their large number of parameters. The ViT base model had 86 million parameters and the ViT large model had 300 million parameters. It is difficult to train from scratch using hundreds or thousands of ultrasound images, as was the case in our study. Therefore, the model, which was trained from scratch, performed poorly. Another observation made from the experiments was that ViTs are more computationally expensive compared to CNNs. Models using ViTs require more time to train owing to the large size of its architecture. Comparing the results in Table 3 and Table 6 in terms of the time taken for training the models, one can observe that CNNs were mostly faster than ViTs to train. Despite the larger number of parameters and higher computational cost compared with CNNs, our proposed ViTs performed better than CNNs in all cases. The transfer-learning method proposed in this study exhibited a superior performance. 

Future work is directed towards optimizing the model using different parameters not included in this paper. Previous studies related to multistage learning methods for ultrasound images have shown the effect of different deep-learning parameters [25,28]. Thus, we will further our efforts in optimizing the proposed model using different deep- learning parameters. Furthermore, we have observed the slight performance difference when using different datasets from different sources, as can be observed in Table 3. The reason for this is obvious and it is due to the difference of imaging equipment, personnel, location, and other related factors. A good deep-learning model should entertain such variations and perform uniformly, irrespective of the source of the dataset. However, this requires the availability of diverse datasets from different locations and in large amounts, which are not currently available. Therefore, our next task will be considering the usage of different datasets across the globe from different locations and training our proposed model. The proposed method could also be translated to breast cancer early diagnosis via other modalities, such as mammogram and magnetic resonance imaging (MRI). Multistage transfer learning using natural images and microscopic images has shown improvement on CNNs’ performance for breast ultrasound image classification [27]. This could also be true for vision transformers’ performance on breast mammogram images. Therefore, the proposed method could be translated to breast cancer early detection using other modalities, such as mammograms. 

## Figures and Tables

**Table 1:** Vision transformer model details.

Model	Layers	Hidden Size	MLP Size	Heads	Patch Size
vitb_16	12	768	3072	12	16 × 16
vitb_32	12	768	3072	12	32 × 32
vitl_32	24	1024	4096	16	32 × 32

**Table 2:** Evaluation metrics.

Metrics	Formula
Accuracy	$\frac{TP+TN}{TP+FP+FN+TN}$
Precision	$\frac{TP}{\left(TP+FP\right)}$
Recall	$\frac{TP}{\left(TP+FN\right)}$
F1 score	$\frac{TP}{TP+\frac{1}{2}\left(FP+FN\right)}$
MCC score	$\frac{TN\times TP-FN\times FP}{\surd \left(TP+FP\right)\left(TP+FN\right)\left(TN+FP\right)\left(TN+FN\right)}$
Kappa score	$\frac{2\times \left(TP\times TN-FN\times FP\right)}{\left(TP+FP\right)\times \left(FP+TN\right)+\left(TP+FN\right)\times \left(FN+TN\right)}$

**Table 3:** The performance of the proposed method: BUSI, breast ultrasound image dataset; LR, learning rate; s, seconds; AUC, area under receiver operating curve.

Model	Optimizer	LR	Accuracy (95%)	AUC (95%)	F1 Score (95%)	Time (s) (95%)	Loss (95%)
Mendeley							
vitb_16	Adam	0.0001	1 ± 0	1 ± 0	1 ± 0	123.35 ± 2.7751	0.33 ± 0.0037
vitb_32	Adam	0.0001	1 ± 0	1 ± 0	1 ± 0	69.85 ± 1.4289	0.33 ± 0.0053
vitl_32	Adam	0.0001	1 ± 0	1 ± 0	1 ± 0	157.16 ± 6.8667	0.33 ± 0.0031
BUSI							
vitb_16	Adam	0.0001	0.952 ± 0.0296	0.968 ± 0.0232	0.966 ± 0.0323	371.39 ± 3.1718	0.68 ± 0.0213
vitb_32	Adam	0.0001	0.944 ± 0.0242	0.958 ± 0.0183	0.942 ± 0.0204	294.874 ± 2.2868	0.72 ± 0.0145
vitl_32	Adam	0.0001	0.928 ± 0.0309	0.947 ± 0.0315	0.922 ± 0.0333	407.074 ± 5.191	0.7± 0.0289
Mixed							
vitb_16	Adam	0.0001	0.919 ± 0.0188	0.937 ± 0.0256	0.919 ± 0.0225	407.024 ± 1.7652	0.43 ± 0.0051
vitb_32	Adam	0.0001	0.904 ± 0.0068	0.929 ± 0.0092	0.904 ± 0.0068	265.434 ± 1.8455	0.45 ± 0.013
vitl_32	Adam	0.0001	0.891 ± 0.0291	0.914 ± 0.343	0.894 ± 0.0335	448.092 ± 5.0215	0.44 ± 0.008

**Table 4:** Performance results of ViT models trained from scratch. BUSI, breast ultrasound image dataset; LR, learning rate; s, seconds; AUC, area under receiver operating curve.

Model	Optimizer	LR	Accuracy (95%)	AUC (95%)	F1 Score (95%)	Time (s) (95%)	Loss (95%)
Mendeley							
vitb_16	Adam	0.0001	0.704 ± 0.3	0.730 ± 0.2	0.706 ± 0.3	123.35 ± 2.7751	0.33 ± 0.0037
vitb_32	Adam	0.0001	0.692 ± 0.1	0.715 ± 0.2	0.693 ± 0.2	69.85 ± 1.4289	0.33 ± 0.0053
vitl_32	Adam	0.0001	0.673 ± 0.1	0.695 ± 0.3	0.671 ± 0.2	157.16 ± 6.8667	0.33 ± 0.0031
BUSI							
vitb_16	Adam	0.0001	0.694 ± 0.0296	0.710 ± 0.07	0.693 ± 0.0323	371.39 ± 3.1718	0.68 ± 0.0213
vitb_32	Adam	0.0001	0.684 ± 0.0242	0.698 ± 0.0183	0.682 ± 0.0204	294.874 ± 2.2868	0.72 ± 0.0145
vitl_32	Adam	0.0001	0.669 ± 0.0309	0.6851 ± 0.0315	0.667 ± 0.0333	407.074 ± 5.191	0.7 ± 0.0289
Mixed							
vitb_16	Adam	0.0001	0.684 ± 0.0188	0.70 ± 0.125	0.689 ± 0.0225	407.024 ± 1.7652	0.43 ± 0.0051
vitb_32	Adam	0.0001	0.674 ± 0.0068	0.699 ± 0.0092	0.68 ± 0.0068	267.434 ± 1.8455	0.45 ± 0.013
vitl_32	Adam	0.0001	0.66 ± 0.0291	0.689 ± 0.343	0.67 ± 0.0335	448.092 ± 5.0215	0.44 ± 0.008

**Table 5:** Performance of conventional transfer-learning-based ViT models. BUSI, breast ultrasound image dataset; LR, learning rate; s, seconds; AUC, area under receiver operating curve.

Model	Optimizer	LR	Accuracy (95%)	AUC (95%)	F1 score (95%)	Time (s) (95%)	Loss (95%)
Mendeley							
vitb_16	Adam	0.0001	1±0	1 ± 0	1 ± 0	118.35 ± 2.7751	0.33 ± 0.0037
vitb_32	Adam	0.0001	1±0	1±0	1 ± 0	64.85 ± 1.4289	0.33 ± 0.0053
vitl_32	Adam	0.0001	1±0	1±0	1 ± 0	152.16 ± 6.8667	0.33 ± 0.0031
BUSI							
vitb_16	Adam	0.0001	0.942 ± 0.0296	0.9541 ± 0.0272	0.936 ± 0.0323	366.39 ± 3.1718	0.68 ± 0.0213
vitb_32	Adam	0.0001	0.934 ± 0.0242	0.9548 ± 0.0183	0.932 ± 0.0204	289.874 ± 2.2868	0.72 ± 0.0145
vitl_32	Adam	0.0001	0.918 ± 0.0309	0.9351 ± 0.0315	0.912 ± 0.0333	402.074 ± 5.191	0.7 ± 0.0289
Mixed							
vitb_16	Adam	0.0001	0.914 ± 0.0188	0.9116 ± 0.0156	0.909 ± 0.0225	402.024 ± 1.7652	0.43 ± 0.0051
vitb_32	Adam	0.0001	0.894 ± 0.0068	0.8799 ± 0.0092	0.884 ± 0.0068	262.434 ± 1.8455	0.45 ± 0.013
vitl_32	Adam	0.0001	0.86 ± 0.0291	0.8499 ± 0.343	0.844 ± 0.0335	443.092 ± 5.0215	0.44 ± 0.008

**Table 6:** Performance of CNN-based transfer-learning models. BUSI, breast ultrasound image dataset; LR, learning rate; s, seconds; AUC, area under receiver operating curve.

Model	Optimizer	LR	Accuracy (95%)	AUC (95%)	F1 score (95%)	Time (s) (95%)	Loss (95%)
Mendeley							
ResNet50	Adam	0.0001	0.965 ± 0.02	0.972 ± 0.01	0.964 ± 0.02	113.35 ± 2.7751	0.33 ± 0.0037
EfficientNetB2	Adam	0.0001	0.961 ± 0.03	0.969 ± 0	0.956 ± 0.03	115.85 ± 1.4289	0.33 ± 0.0053
InceptionNetV3	Adam	0.0001	0.947 ± 0.1	0.951 ± 0.01	0.941 ± 0.02	123.16 ± 6.8667	0.33 ± 0.0031
BUSI							
ResNet50	Adam	0.0001	0.862 ± 0.1	0.879 ± 0.2	0.864 ± 0.2	261.39 ± 3.1718	0.68 ± 0.0213
EfficientNetB2	Adam	0.0001	0.851 ± 0.2	0.864 ± 0.1	0.856 ± 0.3	270.874 ± 2.2868	0.72 ± 0.0145
InceptionNetV3	Adam	0.0001	0.854 ± 0.1	0.87 ± 0.05	0.859 ± 0.1	272.074 ± 5.191	0.7 ± 0.0289
Mixed							
ResNet50	Adam	0.0001	0.838 ± 0.2	0.836 ± 0.08	0.841 ± 0.1	302.024 ± 1.7652	0.43 ± 0.0051
EfficientNetB2	Adam	0.0001	0.824 ± 0.1	0.829 ± 0.1	0.824 ± 0.2	362.434 ± 1.8455	0.45 ± 0.013
InceptionNetV3	Adam	0.0001	0.806 ± 0.07	0.8199 ± 0.1	0.804 ± 0.1	343.092 ± 5.0215	0.44 ± 0.008

## Data Availability

In this study, we used publicly available breast ultrasound images, the Mendeley ultrasound dataset (https://data.mendeley.com/datasets/wmy84gzngw/1 (accessed on 1 July 2022)), and the Breast ultrasound dataset (BUSI) (https://scholar.cu.edu.eg/?q=afahmy/pages/dataset (accessed on 1 July 2022)). Cancer cell line images can be made available upon reasonable requests by contacting the corresponding authors.
